# Orientation of FePt nanoparticles on top of a-SiO_2_/Si(001), MgO(001) and sapphire(0001): effect of thermal treatments and influence of substrate and particle size

**DOI:** 10.3762/bjnano.7.52

**Published:** 2016-04-21

**Authors:** Martin Schilling, Paul Ziemann, Zaoli Zhang, Johannes Biskupek, Ute Kaiser, Ulf Wiedwald

**Affiliations:** 1 Institute of Solid State Physics, Ulm University, 89069 Ulm, Germany; 2 Institute of Surface Chemistry and Catalysis, Ulm University, 89069 Ulm, Germany; 3 Electron Microscopy Group of Materials Science, Ulm University, 89069 Ulm, Germany; 4 Erich Schmid Institute of Materials Science, Jahnstrasse 12, 8700 Leoben, Austria; 5 Faculty of Physics and Center for Nanointegration Duisburg-Essen (CENIDE), University of Duisburg-Essen, 47057 Duisburg, Germany

**Keywords:** FePt, films, high-resolution transmission electron microscopy (HRTEM), nanoparticles, reflection high-energy electron diffraction (RHEED), solid-phase epitaxy, texture

## Abstract

Texture formation and epitaxy of thin metal films and oriented growth of nanoparticles (NPs) on single crystal supports are of general interest for improved physical and chemical properties especially of anisotropic materials. In the case of FePt, the main focus lies on its highly anisotropic magnetic behavior and its catalytic activity, both due to the chemically ordered face-centered tetragonal (fct) L1_0_ phase. If the *c*-axis of the tetragonal system can be aligned normal to the substrate plane, perpendicular magnetic recording could be achieved. Here, we study the orientation of FePt NPs and films on a-SiO_2_/Si(001), i.e., Si(001) with an amorphous (a-) native oxide layer on top, on MgO(001), and on sapphire(0001) substrates. For the NPs of an approximately equiatomic composition, two different sizes were chosen: “small” NPs with diameters in the range of 2–3 nm and “large” ones in the range of 5–8 nm. The 3 nm thick FePt films, deposited by pulsed laser deposition (PLD), served as reference samples. The structural properties were probed in situ, particularly texture formation and epitaxy of the specimens by reflection high-energy electron diffraction (RHEED) and, in case of 3 nm nanoparticles, additionally by high-resolution transmission electron microscopy (HRTEM) after different annealing steps between 200 and 650 °C. The L1_0_ phase is obtained at annealing temperatures above 550 °C for films and 600 °C for nanoparticles in accordance with previous reports. On the amorphous surface of a-SiO_2_/Si substrates we find no preferential orientation neither for FePt films nor nanoparticles even after annealing at 630 °C. On sapphire(0001) supports, however, FePt nanoparticles exhibit a clearly preferred (111) orientation even in the as-prepared state, which can be slightly improved by annealing at 600–650 °C. This improvement depends on the size of NPs: Only the smaller NPs approach a fully developed (111) orientation. On top of MgO(001) the effect of annealing on particle orientation was found to be strongest. From a random orientation in the as-prepared state observed for both, small and large FePt NPs, annealing at 650 °C for 30 min reorients the small particles towards a cube-on-cube epitaxial orientation with a minor fraction of (111)-oriented particles. In contrast, large FePt NPs keep their as-prepared random orientation even after doubling the annealing period at 650 °C to 60 min.

## Introduction

Due to their attractive catalytic properties for oxygen reduction reactions (ORR) [1–2] as well as their high magnetocrystalline anisotropy energy density (MAE), which promises application for next-generation magnetic data storage [3–5], improved fabrication processes of FePt alloy films and nanoparticles (NPs) with approximately equiatomic composition are a prerequisite in pursuit of optimized functionality. Additional attractiveness of this material results from its thermal stability, its corrosion resistance and the possibility to tune its electronic properties [6–7]. Both of the appealing properties, the catalytic as well as the magnetic, hinge on the chemically ordered L1_0_ phase of FePt close to equiatomic composition [6,8]. Though this tetragonal (fct) phase with a reduced lattice parameter *c* of up to 4% is thermodynamically stable at ambient temperature, it is rarely obtained directly due to kinetic barriers. For that reason, as-prepared samples mostly exhibit the high temperature A1 (fcc) structure which has to be transformed into the desired L1_0_ phase by an additional annealing step. Accompanying this structural transformation, one observes highly anisotropic magnetic properties leading to, e.g., huge coercive fields in the Tesla range [4,8–11]. While ultrathin FePt films usually exhibit large magnetic domains, the use of well-separated or at least exchange-decoupled FePt NPs potentially enables the storage of one bit per particle [4,12–13] and the critical particle size for superparamagnetism decreases to 3–4 nm assuming a bulk MAE [5,11]. In a recent experiment, highly (001)-textured FePt-C granular films have been fabricated on MgO buffer layers for heat-assisted magnetic recording. In this experiment, 3–9 nm FePt NPs formed, while C acted as an exchange-decoupling spacer and the NPs reach an imposing MAE of 5.2 MJ·m^−3^, i.e., about 80% of the L1_0_ FePt bulk value [14].

As just mentioned, if FePt films or NPs are prepared at ambient temperature, the specimens usually crystallize in the chemically disordered A1 phase and subsequent annealing at temperatures of 500–700 °C is needed to reach the desired L1_0_ structure [8,15]. For data storage applications, the magnetic easy axis (*c*-axis of the tetragonal crystal) should be aligned perpendicular to the substrate plane [12,16]. Besides other factors, the achievement of specific orientations on a support is also important for catalytic applications since the activity of a specimen can drastically depend on the interplay of NPs with their support [1,17–18].

In the literature, the most-widely used approach leading to epitaxial L1_0_ FePt films with perpendicular magnetization is sputtering or e-beam evaporation on MgO(001) single crystals at elevated temperatures [19] or, alternatively, deposition at ambient temperature followed by an annealing step [20]. On amorphous SiO_2_ or crystalline sapphire(0001) supports, highly (001)-textured FePt films can be fabricated by rapid thermal processing driven by stress-induced reorientation [21], while longer annealing times on the order of minutes lead to reorientation towards (111) texture due to surface energy-driven diffusion [22]. On WSe_2_(0001) supports, epitaxial FePt nanostructures were grown epitaxially with three possible azimuthal orientations [23].

FePt NPs have been prepared by different approaches mainly using colloidal chemistry [5,24], gas-phase preparation techniques [25–26], or thin films of few monolayers under ultrahigh vacuum conditions at elevated temperatures leading to dewetting [23]. Here, we have chosen the so-called micellar approach delivering well-separated and size-tuneable FePt NPs on flat supports [10–11], which is of special interest for the present experiments since particle coalescence, growth or Ostwald ripening by annealing can be completely avoided [15].

In the present study we investigate the possibility of a structural (re)orientation of FePt NPs and thin films on a-SiO_2_/Si(001), MgO(001), and sapphire(0001) after different in situ annealing steps by HRTEM and RHEED. Differently sized sets of NPs were prepared and deposited onto those substrates where they self-assemble into arrays of a high degree of hexagonal order. The FePt thin films served as a reference for solid state epitaxy after similar annealing steps. The above substrates were chosen according to reports in the literature for highly textured L1_0_ thin films [21–22,27]. While HRTEM has proven a reliable experimental technique for the investigation of NPs or thin films in cross-section geometry [20,28] even on an atomic scale, only few reports on the structural investigation of supported NPs by RHEED exist [23,29]. Information provided by these methods is complementary with HRTEM delivering more precise structural details but usually being restricted to a small sample spot. RHEED, on the other hand, can be applied in situ even in between annealing steps and averages the structural information over a much larger area. Such combined information was used here to investigate the orientation of FePt NPs on MgO(001) initiating then further RHEED studies on a-SiO_2_/Si(001) and sapphire(0001) substrates.

## Experimental

FePt NPs were prepared by a micellar technique, which allows for controlling the particle size, distance and arrangement [11]. Since this preparation is almost independent of the substrate, we successfully deposited the well-separated NPs on a-SiO_2_/Si(001), sapphire(0001) and magnesium oxide MgO(001). In brief, reverse micelles were formed using a commercial diblock-copolymer (PS-P2VP) solved in water-free toluene and loaded by Zeise’s salt K[PtCl_3_(C_2_H_4_)]·H_2_O and FeCl_2_ or FeCl_3_ in the appropriate ratio for equiatomic FePt NPs. All chemicals were used as received. A self-assembled close-packed monolayer of precursor-loaded micelles forms on the substrates by dip coating at a typical velocity of 15 mm/min [11,30]. These deposited precursor-loaded micelles are transformed into FePt NPs by a combination of oxygen and hydrogen plasma treatments: The organic shell is removed by oxygen plasma, followed by a subsequent hydrogen plasma step, necessary to completely reduce the NPs into the metallic state. Details on the preparation of NPs can be found elsewhere [11]. Further annealing steps were applied in H_2_ atmosphere at a pressure of 10^−4^ mbar. The plasma etching system is attached to an ultrahigh vacuum chamber (UHV) for structural and chemical analysis allowing in situ inspection by RHEED and X-ray photoelectron spectroscopy (see below). The base pressures in the plasma and the analysis chamber are 5·10^−8^ mbar and 5·10^−10^ mbar, respectively.

a-SiO_2_/Si(001) and sapphire substrates were used as received. For MgO we applied an annealing step at 1100 °C for 12 h in oxygen atmosphere leading to an atomically smooth surface for nanoparticle and film deposition. Since charging of the MgO substrate turned out to deteriorate the RHEED pattern, we further annealed the bare substrate under hydrogen atmosphere at 5·10^−6^ mbar to create oxygen vacancies, leading to a sufficient surface conductivity before dip coating (applied for the small particles). Alternatively, such a surface conductivity was achieved by in situ flash evaporation of a few nanometers of amorphous carbon on top of the already prepared particles directly before RHEED inspection (applied for the larger particles). The carbon film was then completely removed by reactive plasma etching prior to successive annealing steps.

FePt reference films were prepared on native SiO_2_ on top of Si(001), Al_2_O_3_(001), and MgO(001) by pulsed laser deposition (PLD) from Fe and Pt targets under UHV conditions in a separate vacuum system [22,31]. Prior to in situ characterization, the films were reduced by exposure to a hydrogen plasma.

The composition and the chemical state of the FePt NPs and films were measured by X-ray photoelectron spectroscopy (XPS) using Al Kα radiation directly after reduction in hydrogen plasma and after subsequent annealing steps [11,32]. The structure and orientation of FePt NPs and films on the three different substrates were characterized by RHEED at variable electron energies up to 30 keV with an electron beam diameter of about 100 µm. For all RHEED patterns presented in the following, we applied background subtraction using a reference pattern taken with a blanked electron beam, so that only features caused by the light emitted from the filament of the electron gun are visible on the RHEED fluorescent screen.

Following the in situ investigations, ex situ atomic force microscopy (AFM) in tapping mode under ambient conditions is applied for measuring the size distribution of the NPs as well as their spatial arrangement. In this way, possible changes in NP diameter and hexagonal order before and after annealing could be monitored. For that purpose, AFM images were analyzed with the WSxM software [33].

HRTEM studies of cross-section samples are conducted in an aberration-corrected FEI Titan 80-300 microscope at 300 kV. The TEM samples are treated in situ in the same way as RHEED samples and finally a 10–15 nm SiO_2_ capping layer is deposited for oxidation protection prior to standard cross-section preparation via cutting, mechanical grinding and polishing followed by low angle Ar^+^ ion milling.

### Structural analysis of nanoparticles and films on top of single crystalline substrates by electron diffraction in RHEED geometry

In the following, we briefly discuss the RHEED experiments on (i) single crystals, (ii) epitaxial films, and, (iii) the special situation of crystalline NPs on top of single crystalline substrates. In RHEED geometry, the incident electron beam is directed at a low angle to the sample surface. In the case of an ideal spatially unrestricted and atomically flat single crystal, RHEED delivers a pattern reflecting the in-plane symmetry of the surface. Sharp spots occur, arranged in the so-called Laue zones [34]. For thin, pseudomorphously grown films, the spots transform to vertical streaks, with their width being inversely proportional on the lateral dimensions of the crystallites while their length is dominated by the inverse incident angle of the RHEED beam. Additional information can be extracted from the distance between streaks delivering the spacing of the reflecting crystal planes [34].

While the above two experimental situations are well-known from the literature, the RHEED pattern for NPs supported by a flat, single crystal substrate, gets more complex [29]. The expected pattern consist of two contributions: Free substrate areas give the standard Laue zone spot pattern, while for electrons hitting the particles, the experiment turns into transmission electron diffraction being sensitive to the symmetry perpendicular to the beam. In case of randomly oriented NPs, Debye–Scherrer rings appear with the direct beam in the centre of the rings. When the NPs, however, have a preferred orientation perpendicularly to the substrate, the ring structures transform to spot patterns. In our experiments, this fact is used to investigate the reorientation of FePt NPs by subsequent annealing. Note that the known substrate structure can be still used as an intrinsic calibration standard for the orientation and the lattice parameter of the NPs. In addition, the appearance of superstructure {001} and {011} peaks for L1_0_-ordered FePt NPs (as compared to the disordered A1 structure) gives direct proof for the formation of the highly anisotropic L1_0_ phase.

## Results and Discussion

In the following first part we present a HRTEM study of a few FePt NPs on a MgO(001) substrate proving the possibility of NP reorientation on a single crystalline support by post-deposition annealing. This served as motivation for the in situ RHEED investigations of FePt NPs and films on various substrates. The electron spot in the RHEED experiments has a diameter of about 100 µm where the obtained structural information is averaged over more than 10^7^ FePt NPs, thus, as opposed to HRTEM imaging with local structural information of single FePt NPs [8,28,35].

### HRTEM study of FePt nanoparticles on MgO(001)

**Figure 1:**
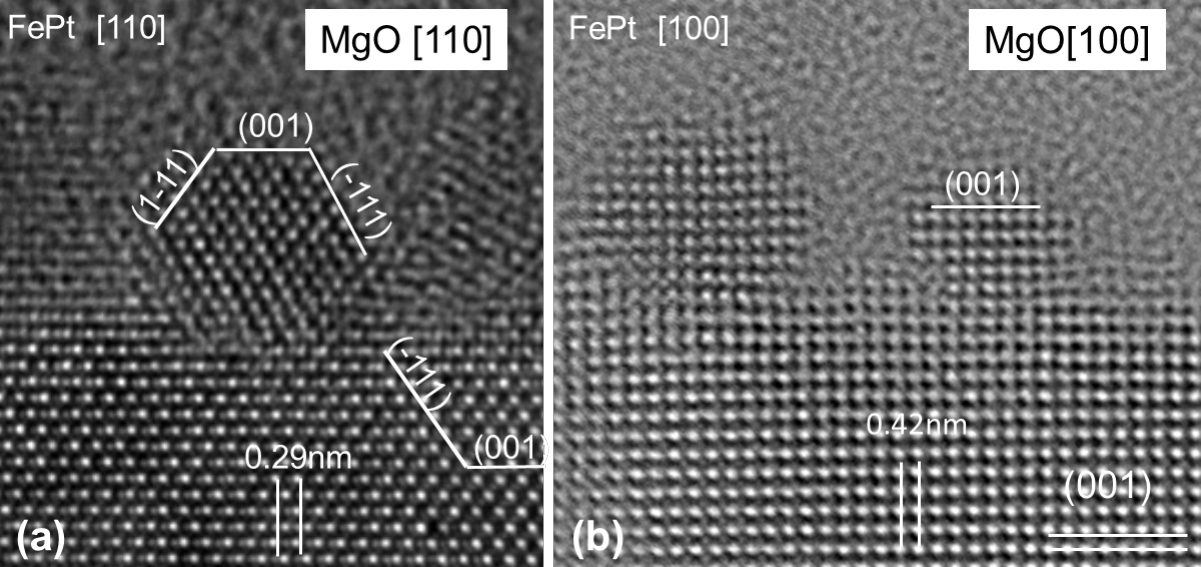
Cross-section HRTEM images of small (2–3 nm) FePt NPs on a MgO(001) substrate after annealing at 650 °C for 30 min: viewed in (a) [110] and (b) [100] direction. In the lower part the MgO substrate with indexed lattice planes is visible and used for calibration. The three FePt NPs in (a) and (b) show clear facets at the surface. The orientation of these small NPs can easily be determined as cube-on-cube epitaxy with FePt(001) || MgO(001) and FePt[100] || MgO[100].

Figure 1 shows HRTEM images of thermally annealed FePt nanoparticles (650 °C for 30 min) on MgO(001) viewed along the [110] and [100] crystal axes of MgO. The epitaxial alignment following a cube-on-cube scheme of the FePt particles on the MgO substrate planes is clearly visible (see indexed lattice planes of FePt and MgO in Figure 1). The FePt nanoparticles exhibit clearly defined facets and are well-bond to the substrate with a special orientation relation. In contrast, prior to annealing even such small FePt nanoparticles did not show a well-defined orientation relationship with MgO substrates, and, in addition, frequently displayed multiple twins.

### FePt nanoparticles and films on SiO_2_/Si(001)

We start the RHEED analysis with experiments on SiO_2_/Si substrates. The about 3 nm thick SiO_2_ serves as a reference substrate, since an amorphous layer should not induce any preferential orientation of deposited NPs or films neither before nor after annealing.

#### Thin FePt films on SiO_2_/Si(001) substrates

**Figure 2:**
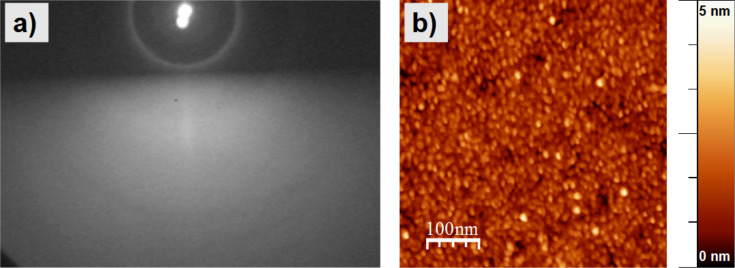
3 nm thick FePt film on SiO_2_/Si(001) substrate after annealing at 650 °C for 30 min: (a) RHEED pattern taken at an electron beam energy of 25 keV delivering just a diffuse intensity distribution without any superposed streaks or spots (the bright ring around the direct beam spot is a halo feature resulting from the RHEED arrangement). (b) AFM image of the FePt film revealing its grainy structure leading to a RMS roughness of 0.6 nm.

In Figure 2a the RHEED pattern for a 3 nm Fe_48_Pt_52_ film on a SiO_2_/Si substrate is presented after post annealing at 650 °C for 30 min. A diffuse intensity distribution is observed without any superposed streaks or spots. The bright ring around the direct beam is a halo feature due to the RHEED arrangement (cf. section Experimental). From the AFM data given in Figure 2b a RMS-roughness of 0.6 nm can be extracted and no evidence for extended flat crystalline domains is found. This is due to the amorphous SiO_2_ layer on top of the supporting Si(001) impeding a substrate-induced pseudomorphic growth. According to this result, a preferred orientation of NPs on Si/SiO_2_ substrates is not expected.

#### FePt nanoparticles on SiO_2_/Si(001) substrate – detection of the L1_0_ phase

**Figure 3:**
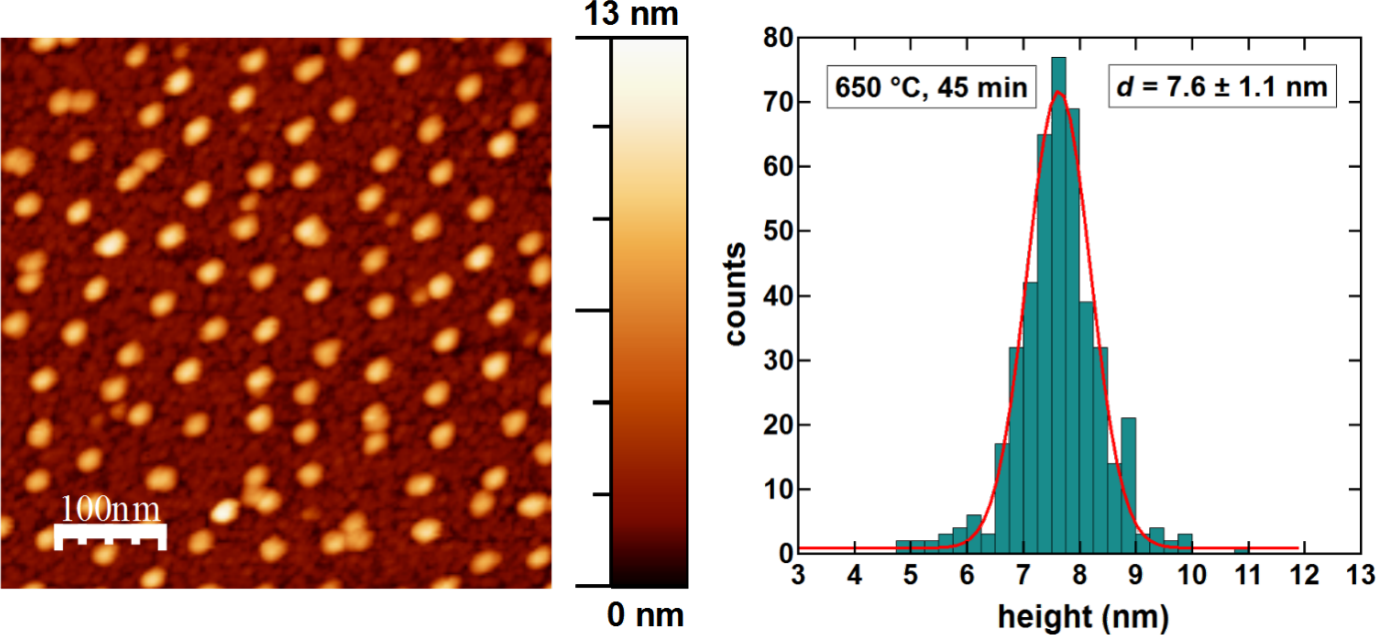
Example of an AFM image (left) and height histogram (right) of micellar FePt NPs on SiO_2_/Si(001) after annealing at 650 °C for 45 min. Compact, well-separated NPs can be observed. The histogram obtained from several AFM images taken at different positions shows a narrow height distribution with an average diameter of 7.6 ± 1.1 nm.

The micellar FePt NPs were prepared on SiO_2_/Si(001) substrates at a slightly Pt-enriched composition of Fe_47_Pt_53_ as determined by XPS (not shown). Details of the XPS analysis are given in [11,32]. For the above composition, the formation of the ordered L1_0_ phase is still expected in bulk samples. To test this for our NPs, they were exposed to a temperature of 630 °C for 45 min. The resulting particle morphology was analysed by AFM (Figure 3) by taking images at different positions on the sample. Compact NPs are found homogeneously distributed over the surface exhibiting a narrow, Gaussian height distribution centred at 7.6 ± 1.1 nm.

**Figure 4:**
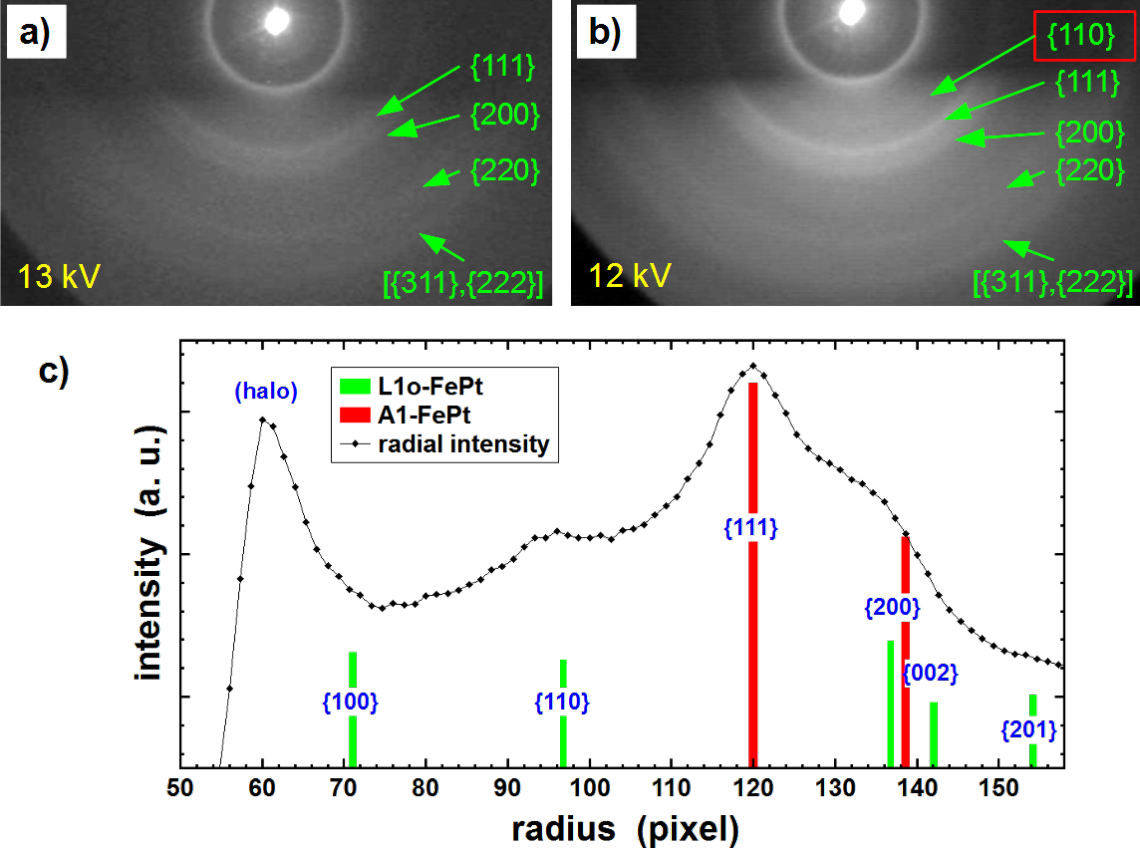
RHEED patterns of 7.6 nm FePt NPs on SiO_2_/Si(001) after different annealing steps (a) 570 °C for 60 min and (b) 630 °C for 45 min. Note the lower electron beam energies of 13 keV and 12 keV, chosen to avoid an overlap of the halo with the {110} ring. The homogeneous Debye–Scherrer rings are used for structural analysis and evaluation of the expected transformation towards the L1_0_ phase. Panel (c) displays the integral of the radial intensity distribution after annealing at 630 °C. The red and green lines indicate the expected positions and intensities of the Debye–Scherrer rings.

The RHEED patterns obtained after an intermediate annealing step at 570 °C for 60 min and after further annealing at 630 °C for 45 min are presented in Figure 4a,b. For that purpose, the electron beam energy was reduced to 12 or 13 keV, respectively, ensuring that the position of the {110} superstructure Debye–Scherrer ring signaling the formation of the ordered L1_0_ phase is beyond the artificial halo close to the center. All detected Debye–Scherrer rings exhibit homogeneous intensity distributions along their periphery confirming the expected isotropic orientation of the particles on top of an amorphous substrate. After annealing at 570 °C for 60 min (cf. Figure 4a), all Debye–Scherrer rings can be assigned to the cubic A1 structure. While a {110} superstructure ring cannot be found, the diffuse intensity distribution due to the amorphous SiO_2_ layer is still visible. A weak {110} ring is, however, observed after a second annealing step at 630 °C for 45 min as shown in Figure 4b. To corroborate this observation, the integral of the radial intensity distribution as displayed in Figure 4c was calculated. The red and green vertical bars mark the radius of the Debye–Scherrer rings and their heights indicate the relative intensities corresponding to the chemically disordered A1 phase and the ordered L1_0_ phase as measured by X-ray diffraction on a powder reference sample [36–37]. Note that X-ray and electron diffraction cannot be directly compared due to the different scattering mechanisms. While the relative peak intensities only allow for a qualitative analysis, the radius of the Debye–Scherrer rings can directly be correlated to X-ray diffraction. The relative ring positions and the position of the direct beam are calibrated by the (111) diffraction ring (highest intensity). At the {110} position, the measured intensity is significantly enhanced over the background. Since the existence of the {110} ring in FePt is a direct proof for chemical order in the L1_0_ phase we conclude that most of the particles were transformed from the A1 into the L1_0_ phase by annealing at 630 °C for 45 min, while the previous step at 570 °C for 60 min was not sufficient to trigger the phase transformation. This finding is also in line with our previous studies on FePt NPs [10].

Up to this point, we have shown that micellar FePt NPs at stoichiometric composition can be prepared on flat substrates such as SiO_2_/Si(001) by plasma processing. These particles are robust to annealing steps up to 630 °C. By such an annealing, however, a significant fraction transforms into the chemically ordered L1_0_ phase. In the following parts, we discuss whether post-preparation annealing steps can lead to preferential orientations of FePt films and NPs on crystalline substrates.

#### FePt nanoparticles and films on sapphire(0001) single crystals

In this section we present RHEED investigations on sapphire(0001) single crystals. For a systematic understanding, we first discuss the orientation of a smooth FePt film, which forms a larger interface with the substrate as compared to the corresponding NPs. Thus, a stronger influence of the substrate to subsequent annealing steps might be expected.

#### FePt films on sapphire(0001)

**Figure 5:**
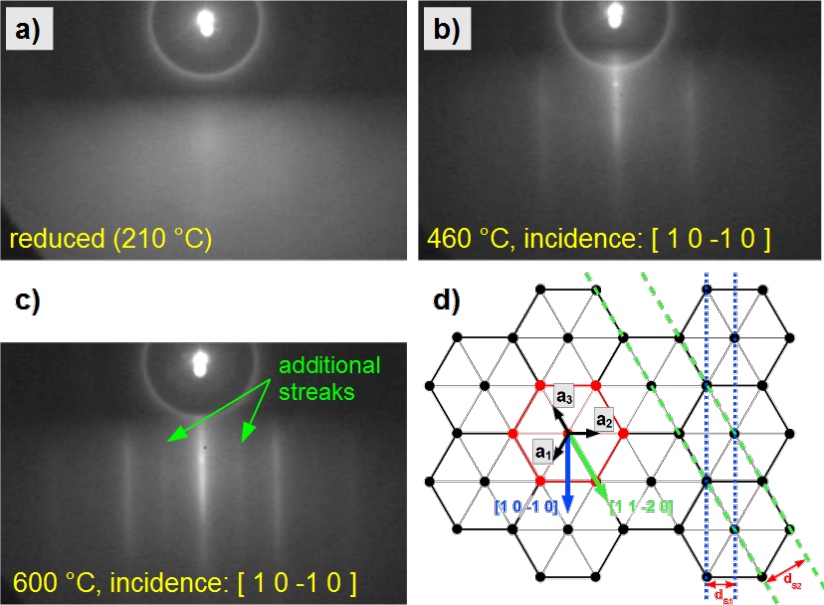
(a–c) RHEED patterns of a FePt film on Al_2_O_3_(0001), recorded with an electron beam energy of 25 keV after different annealing steps at a) 210 °C reduced in hydrogen plasma for 30 min, b) annealed at 460 °C for 30 min, and c) annealed at 600 °C for 45 min. The bright streaks in panel b) and c) indicate highly (111)-textured domains of the cubic A1 phase while the additional weak streaks in c) can be assigned to an additional fraction of the L1_0_ phase with (101)-textured domains. Panel d) illustrates the hexagonal in-plane symmetry of the substrate and the directions of the incident beam.

A 3 nm thick Fe_48_Pt_52_ film was deposited by PLD on sapphire(0001) at ambient temperature. After transfer under ambient conditions, the sample was first reduced in hydrogen plasma at 210 °C for 30 min (see Experimental section) and then characterized in situ for its structural orientation and chemical order. The RHEED image for the as-reduced initial state is presented in Figure 5a showing just a diffuse intensity distribution. Thus, there are no smooth, textured domains present. After subsequent annealing at 460 °C for 30 min and 600 °C for 45 min, symmetric streaks are observed in Figure 5b and Figure 5c, respectively, proving solid phase epitaxy [31]. It should be noted that due to the high surface sensitivity of RHEED and the film thickness of 3 nm, the patterns can exclusively be assigned to the FePt film. On the other hand, azimuthal rotation of the sample with respect to the incident electron beam revealed a hexagonal symmetry and identical patterns are obtained after 60° azimuthal rotation (not shown). Such rotational symmetry corresponds to the symmetry of the sapphire(0001) surface as well as that of a (111)-oriented FePt film. This indicates an epitaxial relation between substrate and the (111)-oriented FePt film. Additionally, by calculating the horizontal separation of the bright streaks in the images of Figure 5b,c from our RHEED geometry, their correlation to the spacing *d*_S1_ of a sapphire(0001) surface is given in Figure 5d. Thus, the electron-beam direction is identified as [10−10]. A further important detail should be noted here. Scrutinizing the pattern of Figure 5c obtained after annealing at 600 °C delivers additional streaks of lower intensity appearing closer to the center. Quantitative analysis of their spacing suggests an assignment to domains in the chemically ordered L1_0_ phase with an (101) orientation relative to the surface, which is in accordance with findings by Ohtake et al. [38] reported for FePd films on MgO(111), a very similar system in view of the symmetry relations. Since the appearance of the RHEED superstructure streaks is in direct relation to the annealing temperature required for the A1→L1_0_ transformation, we exclude that the streaks are related to eventually adsorbed oxygen species. In such case a respective superstructure would already be expected after annealing at 460 °C. Summarizing, FePt films on top of sapphire(0001) annealed at 600 °C for 45 min already exhibit a significant fraction of L1_0_ domains with (101) orientation besides the still dominating (111)-oriented cubic A1 phase. These growth modes agree with those reported in a previous study on FePt and FePd films grown on MgO(111) [39–40].

#### FePt nanoparticles on sapphire(0001) single crystals

**Figure 6:**
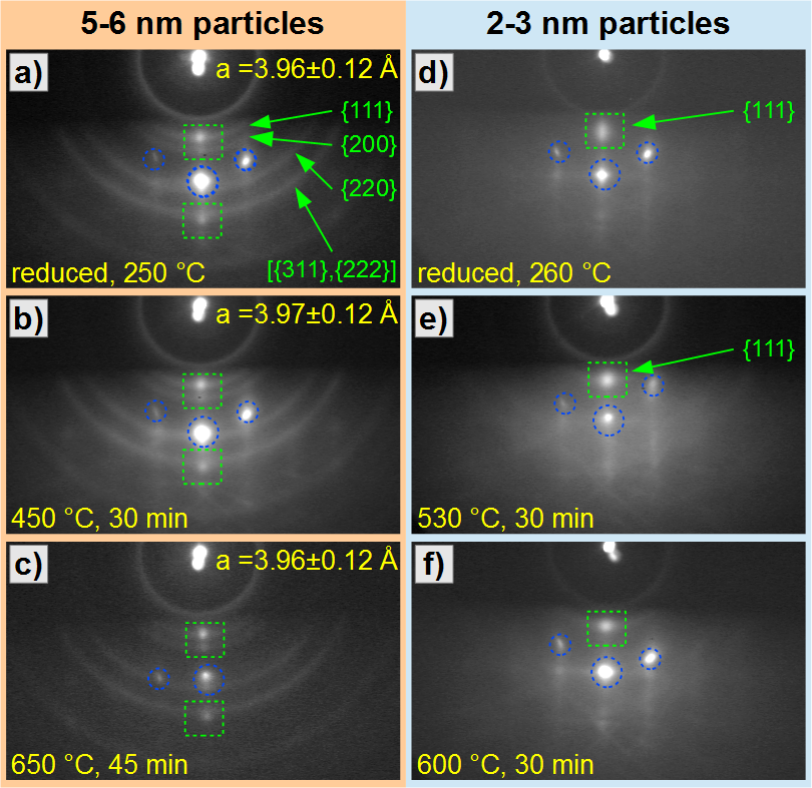
RHEED images of FePt NPs on sapphire(0001) single crystals after several annealing steps, recorded with an electron beam energy of 25 keV. In panels a–c the results for 5–6 nm FePt NPs present clear Debye–Scherrer rings with overlaid (111) and (222) texture spots indicated by green squares. Blue circles mark spots due to reflective diffraction of the sapphire substrate for beam incidence along the [11−20] direction of the sapphire surface. Annealing at 450 °C (b) and 650 °C (c) leads to no significant changes. Panels d–f present the results for 2–3 nm NPs. A dominant (111) spot with a significant vertical elongation is observed after reduction at 260 °C for 20 min. This elongation decreases after annealing at 530 °C (e) and 600 °C (f) indicating improved crystallinity. Debye–Scherrer rings are absent for the 2–3 nm FePt NPs.

The corresponding experiments on the orientation of FePt NPs on top of sapphire(0001) single crystals are presented in Figure 6. The RHEED pattern comprises transmission diffraction from the particles and reflection diffraction from the sapphire(0001) surface as discussed above. For a correct distinction between these two contributions, we exploit the fact that tilting the sample with respect to the incident beam moves the reflective diffraction features vertically, whereas the transmission diffraction features remain at their positions and only display slight intensity changes. The left column of Figure 6 presents RHEED images for 5.1 ± 1.5 nm Fe_47_Pt_53_ particles (“larger” NPs) after several annealing steps (panels a–c). After reduction in hydrogen plasma at 250 °C for 25 min, Debye–Scherrer rings with a weak spot formation (marked by green squares) in the middle of the {111} and {222} rings are observed. This observation already indicates the tendency for a (111) texture of 5.1 nm FePt NPs on sapphire(0001) even in the as-reduced state obtained at 250 °C. The bright centered spot (central blue circle) is due to the specular beam reflection off the sapphire substrate. The two symmetric spots (blue circles left and right) are substrate spots related to the [11−20] in-plane direction and can be used for an in situ distance calibration based on the substrate spacing *d*_S2_ as illustrated in Figure 5d. The lattice parameter *a* of the FePt NPs (A1 structure) was calculated from the (311) diffraction ring delivering *a* = 3.96 ± 0.12 Å. Within the given accuracy, this value is compatible with both, the literature value for A1 FePt *a* = 3.87 Å [37] as well as that for L1_0_ Fe_48_Pt_52_ *a* = 3.86 Å, c = 3.72 Å [8]. Additional annealing steps at 450 °C for 30 min and at 650 °C for 45 min did not significantly change the spot formation on the Debye–Scherrer rings as displayed Figure 5b,c. Similarly, the lattice parameter remains unchanged within the error bar. Thus, we conclude that annealing at temperatures up to 650 °C does not lead to any significant reorientation of the larger FePt NPs on sapphire(0001).

For 2–3 nm (“smaller” NPs) Fe_54_Pt_46_ particles the RHEED images obtained after similar annealing steps as above are presented in the right column of Figure 6. After reduction in hydrogen plasma at 260 °C for 20 min a very intense (111) texture spot is observed as indicated in panel d) by the green square. The extension of this feature is considerably larger than for the 5–6 nm FePt NPs in panel a). Debye–Scherrer rings of larger radii are absent. The bright spots marked by blue circles belong to the sapphire substrate as above. After annealing at 530 °C for 30 min and 600 °C for 30 min, the vertical width of the (111) feature sharpens, whereas no significant change in the other parts of the pattern is observed. The absence of particle coalescence induced by annealing is verified by AFM after the RHEED measurements (not shown).

The relatively strong (111) spot indicates an almost complete (111) orientation of these smaller NPs already after the reduction step at 260 °C. Since the intensity and the width of the spot depends on the degree of (111) orientation and the crystallite size, the observed sharpening after annealing at higher temperatures suggests an improvement of the crystallinity and orientation. For such strong preferential (111) orientation, the intensity contributions from other planes are expected to vanish at the centered position of the pattern. Indeed, we cannot detect any Debye–Scherrer rings or off-centered spots related to the particles. Due to the relatively high background intensity and the small particle size in the patterns of Figure 6d–f, however, it cannot be completely excluded that a low residual intensity of other lattice planes still remains.

In summary, these experiments indicate the general existence of a (111) preferential orientation of FePt NPs on sapphire(0001). While larger NPs, however, exhibit just a tendency for (111) texture formation, the smaller NPs appear strongly oriented already in their as-reduced state at 260 °C.

#### FePt nanoparticles and films on MgO(001) single crystals

Successful growth of highly oriented L1_0_ FePt thin films on top of MgO(001) has already been reported previously [19–20]. Similarly, for FePt nanoparticles on MgO(001) the above HRTEM investigation proved the possibility of annealing induced epitaxial order at least in a few cases for particles with diameters smaller than 3 nm (cf. Figure 1). Here, we are testing whether (i) the majority of the particles can be epitaxially oriented and (ii) this observation holds for particles larger than 3 nm. For both, FePt films and particles on MgO(001), the observed cube-on-cube growth mode appears plausible due to the 9% lattice mismatch between the two systems leading to pseudo-epitaxial growth with 11 FePt lattice parameters fitting well onto 10 lattice units of a MgO(001) substrate [20]. RHEED measurements are first performed on a thin FePt film after annealing serving as reference before we focus on 2–3 nm and 5–6 nm FePt NPs.

#### FePt films on MgO(001) single crystals

**Figure 7:**
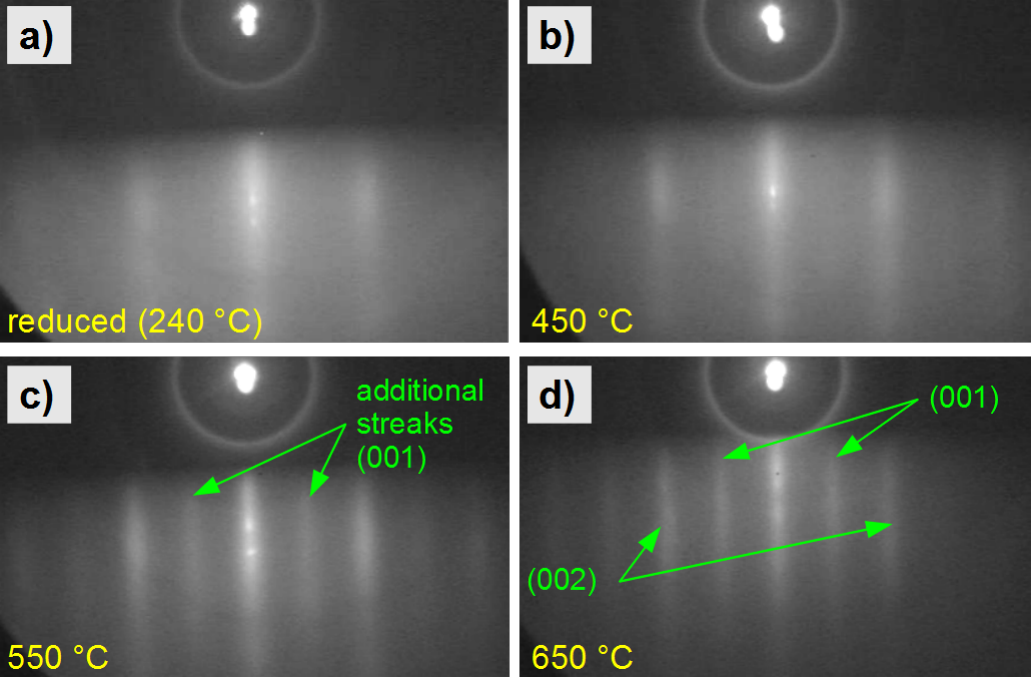
(a–d) RHEED pattern of a FePt film on MgO(100), recorded with an electron beam energy of 25 keV after several annealing steps (each for 30 min) measured under a [100] direction of the incident beam relative to the surface. (a) Broad streaks indicating texture are already visible for the as-reduced film. (c) Additional streaks appear by annealing at 550 °C indicating transformation into the chemical ordered L1_0_ phase of the textured domains. (d) Streaks sharpen by annealing at 650 °C.

In this paragraph, we discuss the structural orientation and the chemical phase of a 3 nm thick FePt film on MgO(001). The Fe_48_Pt_52_ film was prepared by PLD at ambient temperature. The RHEED pattern after reduction at 240 °C for 30 min in a hydrogen plasma is presented in Figure 7a already revealing the formation of RHEED streaks. This indicates the existence of smooth textured domains and a low surface roughness. After subsequent in situ annealing steps of 30 min at 450, 550 and 650 °C the width of the streaks decreases as demonstrated in Figure 7b–d. After annealing at 550 °C, the appearance of superstructure streaks is detected, which become even sharper after annealing at 650 °C. RHEED images taken after in-plane rotation of the film allowed determining the incident beam direction as [100] within the (001) plane of the sample. With this relation one can assign the separation of the brightest streaks to the distance of the (002) planes and the separation of the additional streaks to the distance of the (001) planes. This is a clear indication that the film transformed into the L1_0_ phase by annealing at 550 °C with improved perfection of this phase at even higher temperatures. Its *c*-axis, however, has to be oriented within the surface plane, since the (001) diffraction signal is forbidden for the A1 phase. Any decision on the existence of L1_0_ domains with their *c*-axis perpendicular to the film plane is impossible by using RHEED, since due to symmetry and instrument resolution we cannot distinguish them from A1 domains. Similar arguments have been used by Ohtake et al. identifying the L1_0_ phase with an in-plane *c*-axis for a FePd film on MgO(001) [40]. Due to the high temperature required to obtain the RHEED L1_0_ superstructure streaks we conclude that adsorbed oxygen species are very unlikely to be their reason, since those should already have formed at the lower annealing temperature (450 °C).

#### FePt nanoparticles on MgO(001) single crystals

**Figure 8:**
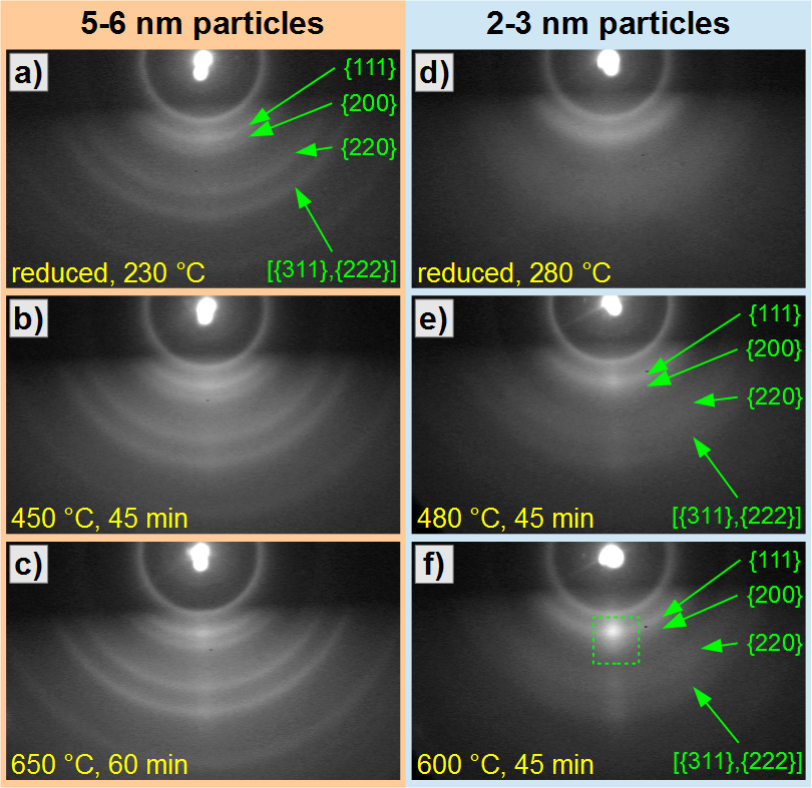
(a–c) RHEED patterns of large (5–6 nm) FePt nanoparticles prepared on MgO(001), recorded with an electron beam energy of 25 keV after different annealing steps. The patterns show Debye–Scherrer rings with a homogeneous intensity along the ring periphery indicating a random orientation of the nanoparticles. By annealing at 650 °C the sharpness of the rings is slightly enhanced. (d–f) RHEED patterns of small (2–3 nm) FePt nanoparticles prepared on MgO(001) after several annealing steps. Broad Debye–Scherrer rings appear due to the small particle size. (d) After reduction, a homogeneous intensity indicates a random particle orientation. Subsequent annealing at 480 °C (e) leads to a weak and at 600 °C (f) to a strong spot formation (green square) indicating a preference for (111) and (001) orientations.

The orientation of as-reduced as well as subsequently annealed FePt NPs on MgO(001) has been tested for particle batches with diameters of 5–6 nm and 2–3 nm, identical particles as for the RHEED experiments above. Panels a–c of Figure 8 present the RHEED images for the larger 5–6 nm Fe_47_Pt_53_ NPs on MgO(001) after reduction at 230 °C for 30 min (a), and after annealing steps at (b) 450 °C for 45 min and (c) 650 °C for 60 min. After reduction in hydrogen plasma, clearly distinguishable Debye–Scherrer rings are observed, which can easily be indexed by assigning to the corresponding lattice planes. In this experiment, charging has been minimized by a carbon flash evaporation, which, however, is removed by plasma etching before the subsequent annealing steps. The intensity along the Debye–Scherrer rings is homogeneous, thus the particles are randomly oriented. Diffraction features from the substrate are not visible. After annealing at 450 °C (panel b) no significant changes of the RHEED pattern could be observed. After an additional annealing step at 650 °C, the rings get sharper (panel c). The slightly enhanced intensity around the center of the rings is attributed to the central RHEED streak of the substrate, since it is overlaying all rings in the same way. The absence of any spot formation on the Debye–Scherrer rings (as observed for the identical particles on sapphire(0001)) is a clear indication, that even after annealing at 650 °C it is not possible to induce a preferential orientation of larger, 5–6 nm FePt particles on MgO(001). The enhanced sharpness of the rings can be attributed to an improved crystallinity of the nanoparticles after annealing at 650 °C, but also to decreasing charging effects due to the formation of surface defects in the MgO substrate leading to better electrical conductivity [41]. The absence of substrate spots in the patterns can be explained by the cubic structure of MgO, for which the low-indexed RHEED spots are forbidden. The second order spots have a larger separation, and a lower intensity. In a reference measurement on a bare MgO(001) we further found those spots to appear as streaks (not shown) and therefore with a strongly reduced intensity as compared to sapphire(0001) supports. These findings point to a lower surface quality of the MgO(001) substrate as compared to sapphire(0001). Separate AFM investigations also revealed a narrower width of terraces for the MgO surface than for the sapphire support (not shown).

**Figure 9:**
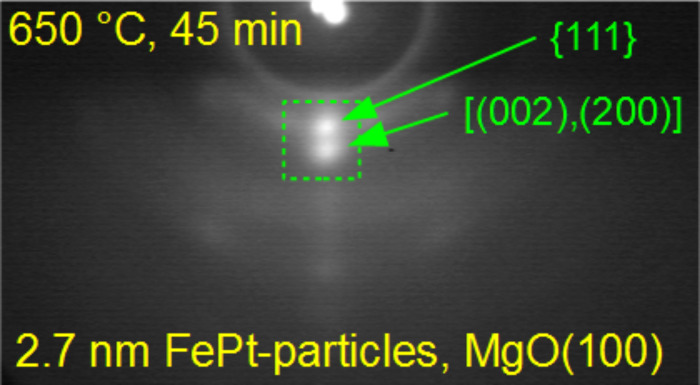
RHEED pattern of 2–3 nm FePt particles on MgO(001), recorded with an beam energy of 25 keV after annealing at 650 °C for 45 min clearly shows texture formation with spots on the (111) and (200), (002) Debye–Scherrer rings.

Further, we investigated 2–3 nm Fe_54_Pt_46_ particles as shown in RHEED patterns after (Figure 8d) reduction at 280 °C for 30 min, and after annealing at (Figure 8e) 480 °C for 45 min and (Figure 8f) 600 °C for 45 min. For the smaller particles, we also find Debye–Scherrer rings with a homogeneous intensity distribution along their peripheries exhibiting, however, an increased width due to the small particle size. By annealing at 480 °C the pattern sharpens and a slightly enhanced intensity around the center of the {111} ring is observed. After further annealing at 600 °C a diffraction spot appears as highlighted by the green square in Figure 8f providing evidence for a thermally induced re-orientation of the smaller FePt NPs. It is evident, that the spot intensity increases not only at the center of the {111} ring but also of the {200} ring pointing to an additional though weaker (100) or (001) orientation. To corroborate this trend, we directly annealed a second, independent sample with identical nanoparticles on MgO(001) at 650 °C for 45 min after hydrogen plasma reduction. The respective RHEED pattern is shown in Figure 9. We observe a clear spot formation in the center of the {111} and (200)/(002) Debye–Scherrer rings. This confirms the thermally induced reorientation of smaller FePt NPs by annealing at 650 °C resulting in a mixed (111) and (001) texture. More quantitatively, the intensity ratio of the (200) with respect to the (111) spot in Figure 9 is determined to approximately 0.9 by evaluating the integral intensities. The still present (111) orientation may be caused by different surface energies leading to the expected Wulff shape of the small particles with favored (111) facets [28,42–43], while the (100) orientation is induced by the MgO(001) substrate as suggested by results from film growth [19]. Further, the brighter off-centered areas at the {220} and {222} rings have rather to be attributed to the fact that at these positions an overlap occurs of the respective (rather weak) Debye–Scherrer rings and vertical RHEED streaks of the MgO(001) substrate. This fact was checked by tilting the sample and varying the angle of incidence with respect to the surface. By this, the substrate features were changing their position very sensitively with the tilt, whereas the transmission features of the particles kept their position.

**Figure 10:**
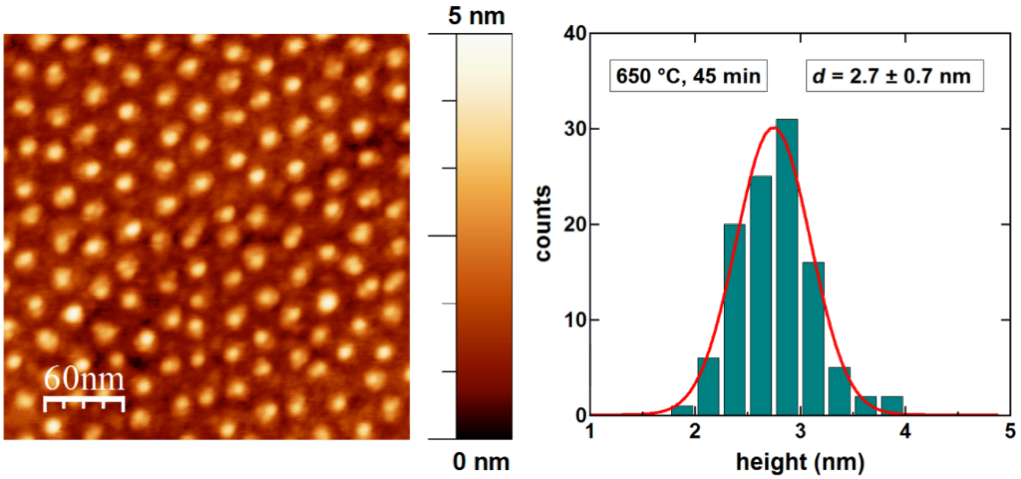
AFM image and height histogram of 2–3 nm FePt nanoparticles on MgO(001) after annealing at 650 °C for 45 min. The particles are well separated in a hexagonal arrangement. The height histogram shows the size distribution centered at 2.7 nm.

In order to analyze the morphology of the smaller FePt NPs after the above heat treatment at 650 °C, subsequent AFM measurements were performed delivering the data presented in Figure 10. The AFM image clearly proves the high degree of hexagonal order of the particle arrangement as well as the absence of any particle coalescence during the annealing up to temperatures of 650 °C. The height distribution of the nanoparticles (right panel of Figure 10) yields an average value of 2.7 ± 0.7 nm identical to the as-prepared value. Thus, thermal evaporation of the FePt NPs during annealing at 650 °C due to a possible size-dependent vapor pressure enhancement can be excluded.

## Conclusion

We studied, by using HRTEM and RHEED, the orientation of FePt NPs on a-SiO_2_/Si, MgO(001), and sapphire(0001) supports after reduction in hydrogen plasma and subsequent annealing steps at temperatures up to 650 °C. For all substrates, additional RHEED experiments on FePt films served as a reference. Two classes of well-separated FePt NPs were prepared by a reverse micelle approach: “large” ones within the diameter range of 5–8 nm and “small” ones within 2–3 nm. The micellar preparation route with its control over the interparticle distance guaranteed to avoid any coalescence or Ostwald ripening even after repeated annealing steps at high temperature as demonstrated by related AFM measurements. It is found that the structural and chemical differences of the various substrate surfaces influence the orientation distribution in the as-prepared state of reduced FePt NPs as well as in their state after subsequent annealing. In addition, it turned out that in case of MgO(001) a reorientation from randomly oriented into highly textured NPs can be thermally induced. In that case, however, the reorientation by annealing strongly hinges on the particle size with a restriction to small NPs. A summary of the particle orientation distributions and their response to annealing treatments is presented in Table 1.

**Table 1:** Experimental results of the preferred orientations of FePt nanoparticles on various substrates after particle reduction and in situ annealing. The last column summarizes the improvement regarding crystallite growth and reorientation.

substrate	particle size	orientation after reduction at 210–280 °C	orientation after annealing at 600–650 °C	qualitative influence of annealing (600–650 °C)

a-SiO_2_/Si(001)	7–8 nm	none	none	improved crystallinity
sapphire(0001)	2–3 nm	(111)	(111)	improved crystallinity
	5–6 nm	(111)	(111)	improved crystallinity
MgO(001)	2–3 nm	none	(001), (111)	improved crystallinity and particle reorientation
	5–6 nm	none	none	improved crystallinity

On amorphous SiO_2_/Si(001), 7–8 nm FePt NPs are randomly oriented as-prepared as well as after annealing at 630 °C for 45 min. Such an annealing, however, transforms the particles into the chemically ordered L1_0_ phase as proven by (110) Debye–Scherrer rings forbidden in the A1 phase.

On top of sapphire(0001) substrates FePt particles, i.e., small 2–3 nm and large 5–6 nm NPs, exhibit a fractional (111) texture already in the as-reduced state as opposed to a 3 nm thick FePt film, which shows such a texture only after annealing at 460 °C for 30 min. By further annealing, this texture can be clearly improved because the symmetry of the surface-energy-favored (111) FePt facets matches the hexagonal symmetry of the (0001) sapphire surface. In case of NPs, the thermally induced texture improvement, however, exhibits a size effect: Only the smaller NPs approach a fully developed (111) texture as indicated by the absence of any Debye–Scherrer rings, while for the larger NPs this texture, though dominant, is note complete. It is further worth noting that for all samples, film and NPs, annealing at 600 °C for 30 min leads to L1_0_ ordering.

For FePt NPs on MgO(001) substrates the RHEED analysis was complemented by HRTEM measurements on selected particles. While RHEED demonstrated that both, small and large FePt NPs are randomly oriented in their as-reduced state, the small ones exhibit a surprising re-orientation after annealing at 650 °C for 30 min towards a dominating (001) orientation with an admixture of (111) oriented NPs. The preferred cube-on-cube epitaxial relation for 3 nm FePt nanoparticles after annealing at 650 °C for 30 min was convincingly confirmed by HRTEM. Opposed to this thermally induced re-orientation of small NPs, for the larger particles with diameters in the 5–6 nm range annealing at the identical temperature of 650 °C even for a doubled period of 60 min did not lead to a change of their random orientation. At this point, it is worth adding a general remark on the competition between surface-energy-related driving forces favoring certain facets during NP growth implying corresponding rotational symmetries and forces that are related to the particle–substrate interface with corresponding substrate-induced symmetries. In the present case of FePt NPs, surface energies prefer (111) facets with their symmetry being compatible with the hexagonal symmetry of sapphire(0001), while being incompatible with the 4-fold symmetry of MgO(001). As a consequence, on sapphire even the as-prepared FePt NPs exhibit a (111) orientation, which is preserved by annealing and, for small NPs is even complete. On MgO(001), however, the symmetry mismatch leads to a random orientation for as-prepared NPs and only small particles experience a substrate enforced re-orientation towards (001), but still with a residual fraction of (111) oriented NPs. Finally, on an amorphous substrate such as the SiO_2_ surface of a Si crystal, the orientation of as-prepared FePt NPs is random and remains like that for all NP sizes even after annealing.
